# Oxygen reduction reaction on Pt-skin Pt_3_V(111) fuel cell cathode: a density functional theory study

**DOI:** 10.1039/d0ra02972f

**Published:** 2020-07-21

**Authors:** Asnake Sahele Haile, Weldegebriel Yohannes, Yedilfana Setarge Mekonnen

**Affiliations:** Center for Environmental Science, College of Natural and Computational Sciences, Addis Ababa University P.O. Box 1176 Addis Ababa Ethiopia yedilfana.setarge@aau.edu.et; Chemistry Department, College of Natural and Computational Sciences, Addis Ababa University P.O. Box 1176 Addis Ababa Ethiopia

## Abstract

Pt-non-precious transition metals (Pt-NPTMs) alloy electrocatalysts have gained considerable attention to develop cheaper and efficient electrocatalysts for oxygen reduction reaction (ORR) in proton exchange membrane fuel cells (PEMFCs). In this report, density functional theory (DFT) has been applied to study the catalytic activity of Pt-skin Pt_3_V(111) electrocatalyst for ORR in PEMFCs. The results revealed that the ORR intermediates (O, OH and OOH) have lower binding energies on Pt-skin Pt_3_V(111) compared to pure Pt(111) surface. The ORR on Pt-skin Pt_3_V(111) surface proceed *via* OOH dissociation with an activation energy of 0.33 eV. The formation of OH is found to be the rate determining step with an activation energy of 0.64 eV, which is even lower than in pure Pt(111) surface (0.72 eV). This indicates a better performance of Pt-skin Pt_3_V(111) for ORR compared to pure Pt(111) surface. Moreover, the DFT results revealed that the negative formation energy of the Pt_3_V alloy and the positive dissolution potential shift of the surface Pt atoms revealed the better stability of Pt-skin Pt_3_V(111) surface over pristine Pt(111) surface. Due to the improved activity and better stability, the new Pt_3_V alloy electrocatalyst is very promising for the development of low-cost and efficient PEMFCs.

## Abstract 2

Pt-non-precious transition metals (Pt-NPTMs) alloy electrocatalysts have gained considerable attention to develop cheaper and efficient electrocatalysts for oxygen reduction reaction (ORR) in proton exchange membrane fuel cells (PEMFCs).

## Graphical abstract

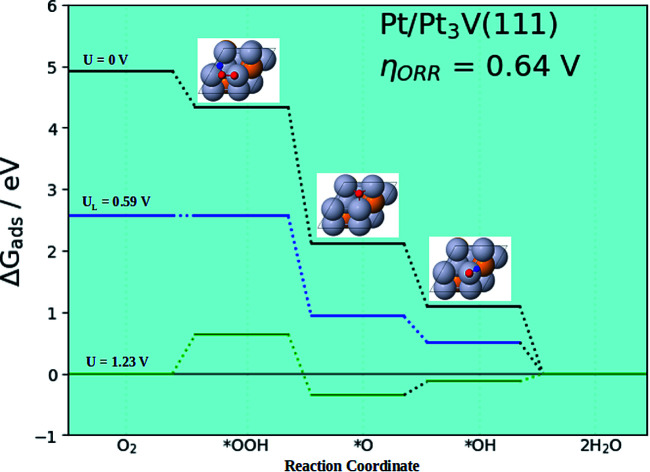

## 1 Introduction

The search for economically and environmentally sustainable energy sources is vital to meet the growing global energy demand. The continuous depletion of fossil fuels and the negative impact of carbon dioxide emissions on the environment have stimulated the development of alternative clean energy technologies from renewable energy sources.^1^ Hydrogen is a promising clean energy carrier with the potential to replace or reduce the reliance on hydrocarbon fuels and thermomechanical engines. Due to these advantages, hydrogen is an ideal fuel for developing efficient energy conversion systems such as fuel cells.

Fuel cell technologies such as proton exchange membrane fuel cell (PEMFC) uses platinum as catalyst to accelerate the hydrogen oxidation reaction (HOR) on an anode and the oxygen reduction reaction (ORR) on a cathode.^2–7^ ORR is one of the key steps in PEMFC. However, the sluggish kinetics of ORR and expensiveness of Pt-based catalysts have become major obstacles towards achieving efficient and cheaper PEMFCs.^8–10^ This led to an active search for economically viable electrocatalyst with high catalytic activity. Alloying platinum with non precious transition metals (NPTMs) such as Ni, Co, Fe, Ti and V is among the promising solutions to enhance the catalytic activity for ORR and reduce the cost.^11–15^ This is because combination of Pt with NPTMs enable to modify the electronic properties in a way that catalytic activity could be enhanced. In addition, replacing pure Pt with Pt alloys reduces Pt loading in PEMFC electrodes.^16^ Previous study by Mukerjee *et al.*^17^ reported that improvement in electrocatalytic activity has been shown by alloying Pt with the first row transition metals (Cr, Mn, Fe, Co and Ni). Recent study by Toda *et al.*^18^ have showed that the alloying approach is efficient in reducing Pt metal loading, without affecting catalytic activity for ORR. This group achieved maximum catalytic activity for 30, 40 and 50% content of Ni, Co and Fe, respectively, by which 10, 15 and 20 times larger kinetic current densities compared to that of pure Pt.

One reason for the exact origin of the ORR activity enhancement of Pt-NPTMs alloy could be the shortening of Pt–Pt inter-atomic distance (*i.e.* structural effect).^19,20^ Alloying Pt with NPTMs of smaller atomic size is believed to decrease the inter-atomic distance between the Pt atoms because of lattice contraction and provide favorable sites for adsorption of molecular oxygen. Min *et al.*^20^ prepared bimetallic alloys of Pt with Co, Cr, or Ni and employed EXAFS (extended X-ray absorption fine structure) analysis to determine the Pt–Pt neighbouring distances. They found nearly linear increase in specific activity with decreasing Pt–Pt bond distance of the catalysts. Furthermore, EXAFS studies indicated variations in electronic states upon alloying Pt with the transition metals.

The other reason for ORR activity improvement of Pt-NPTMs alloy could be due to an increment of Pt d-band vacancy (*i.e.* electronic effect).^21^ Increased d-band vacancy is believed to strengthen Pt–O_2_ bond and thus facilitating O–O bond cleavage. Toda *et al.*^18^ proposed that the increased d-band vacancy in the Pt atoms leads to an increased 2π electron donation from O_2_ to the surface Pt. This resulted in stronger Pt–O bond and a weaker O–O bond. Stamenkovic *et al.*^11^ studied the relationship between experimentally determined surface electronic structure (the d-band center) and the ORR activity of some Pt_3_M (M = Ni, Co, Fe, Ti, V) surfaces. The correlation exhibits “volcano-type” behavior which means that for the optimum catalytic activity there must be a balance between adsorption strength of reactive intermediates and the ability to dissociate surface oxygen species. The adsorption and dissociation properties depend on the valence electronic structure of the metal, specifically, the density of states near the Fermi level.^22^ A shifting of the metal d states upward relative to the Fermi level results in a strong metal–oxygen bond facilitating cleavage of O–O bond. On the other hand, if the d-band center is too close to the Fermi level, the strongly adsorbed intermediates limit the availability of free metal sites.

The Pt monolayer structures are promising ORR catalysts for the reduction of high Pt content and for catalytic activity improvement of a conventional Pt or Pt-based alloys.^11,23–27^ It should be noted that Pt–V system have been attracted many attentions because of its excellent electronic effect and better catalytic activity for oxygen reduction reactions and methanol oxidation. For instance, in 1986 Pt–V alloy was first applied as a catalyst by Cambanis and Chadwick,^14^ and later Antolini *et al.*^28^ investigated the ORR activity of Pt–V alloy and reported an improved performance. Ang and Walsh^29^ synthesized a Carbon black (Vulcan XC72R)-supported Pd–V electrocatalyst using wet chemical reduction of metal chloride salts, which exhibits higher catalytic activity for ORR. Recently, Zhang *et al.*^30^ prepared a Pt–V alloy nanonetwork (ANN)/multiwalled carbon nanotube, which exhibits excellent electrocatalytic performance in both activity and stability for the methanol oxidation reaction (MOR). In addition, DiSalvo *et al.*^31^ successfully synthesized a structurally ordered Pt_3_V alloy by employing a surfactant-free method in an aprotic solvent (THF), the as obtained electrocatalyst material have both enhanced activity and stability for MOR over that of pure Pt catalysts. To the best of our knowledge, there is no computational study report on ORR mechanism of Pt-skin Pt_3_V(111) alloy electrocatalyst for PEMFCs.

Therefore, in this paper, we investigated the ORR mechanism on Pt-skin Pt_3_V(111) and the stability of proposed Pt-skin Pt_3_V(111) alloy catalyst in PEMFC. Thermodynamically optimized Pt-skin Pt_3_V(111) surface model with four layers has been considered. The first layer (top surface) is fully platinum and the other three layers consist of Pt_3_V, 3 : 1 ratio of Pt and V atoms. Density functional theory (DFT) is employed to estimate the adsorption energies of ORR intermediates (O, OH and OOH) and activation energies (*E*_a_) of the elementary reactions of ORR on Pt-skin Pt_3_V(111). In addition, based on the calculated dissolution potential shift (Δ*U*) and alloy formation energy, stability of the electrocatalyst was evaluated.

## 2 Computational details

In this study, spin polarized DFT calculations were performed as implemented in the Vienna *Ab initio* Simulation Package (VASP)^32–35^ integrated with Atomic Simulation Environment (ASE).^36^ The projector augmented wave (PAW) method^37,38^ is used to describe the core electrons, and the basis set for the electronic wavefunctions are plane waves below a 400 eV energy cutoff. Fermi smearing of electronic occupations with a width of 0.1 eV was employed throughout the calculations. 5 × 5 × 1 Monkhorst–Pack *k*-point mesh was used to sample the Brillouin zone.^39^ Revised Perdew–Burke–Ernzerhof (RPBE) exchange–correlation functional was employed,^40^ which provides better chemisorption energies than PBE. All structures were optimized when the Hellmann–Feynman forces on each ion are less than 0.01 eV Å^−1^.

**Fig. 1:**
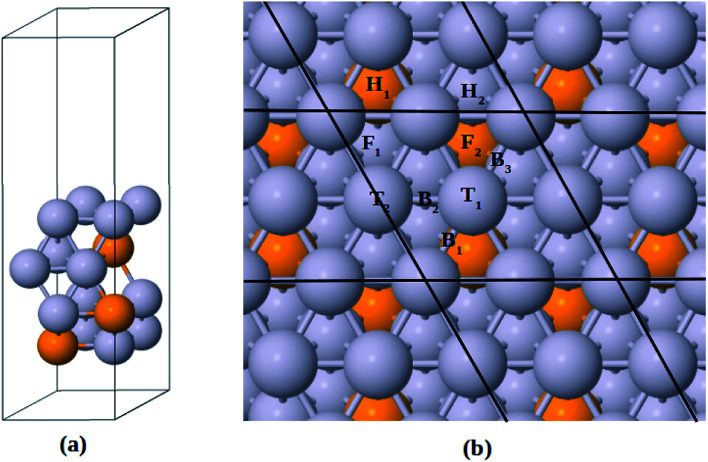
(a) Side view for the slab model of Pt-skin Pt_3_V(111) surface. (b) Adsorption sites on Pt-skin Pt_3_V(111) surface. Pt and V atoms are labeled in gray and orange, respectively.

The Pt-skin Pt_3_V(111) alloy surface is modeled as a pure Pt outermost layer with L1_2_-ordered Pt_3_V crystal structure as the core. Similar previous studies have also reported that ordered Pt_3_V belongs to the binary intermetallic alloy which can crystallize in L1_2_ crystal structure phase^42–44^ in which three Pt atoms placed at the face center positions and one V atom occupies the corner (origin) position is the stable crystal structure. The lattice parameters for Pt and Pt_3_V alloy were calculated and the results were found to be 3.9942 Å for Pt which was in a good agreement with the previous reports,^45^ and 3.93 Å for Pt_3_V which was also agrees with the previous theoretical results.^44,46,47^ Our Pt_3_V(111) surface model was represented by 2 × 2 supercell that periodically repeated in three dimensions with four atoms per cell and four layers of atoms (Fig. 1a). In order to prevent the artificial interactions between the slab and its images, a 14 Å-thick vacuum was added along the direction perpendicular to the surface. In all of the structure optimization calculations, the atoms in the bottom two layers are fixed while all the other atoms are fully relaxed. Solvation of the surface and adsorbates is taken into account by using the implicit solvation model implemented in VASPsol.^48,49^ VASPsol, due to its simplicity and low computational costs, has been applied in different electrochemical systems in recent years.^50–56^ The dielectric constant was set to be 78.54 for H_2_O solvent.

The adsorption energies (Δ*E*_ads_) of ORR intermediates are defined as the DFT energies of the following reactions:

1 * + H_2_O → O* + H_2_,  Δ*E*_ads_(O*)

2
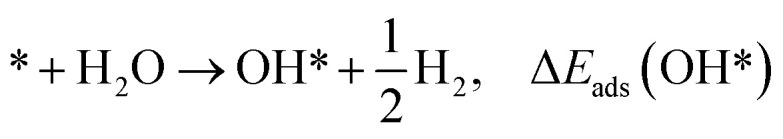
3
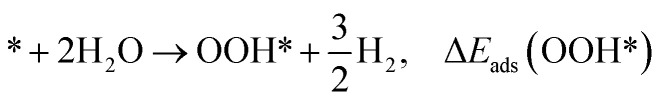
4
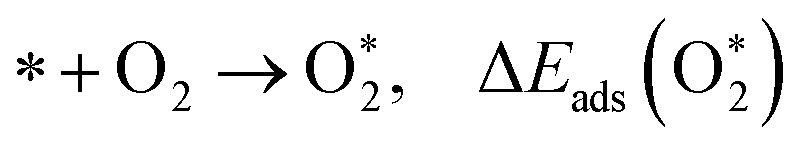
where * denotes the active site on the surface of the catalyst. Thus, the Δ*E*_ads_ of the ORR intermediates can be calculated as,

5 Δ*E*_ads_(O*) = *E*_O*_ − E_*_ − (*E*_H_2_O_ − *E*_H_2__)

6
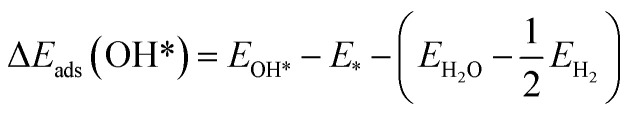
7
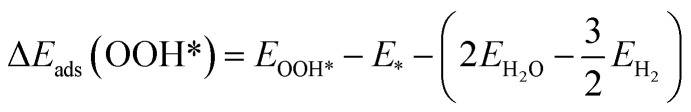
8
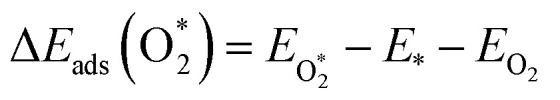


The reaction energy (*E*_r_) and activation energy (*E*_a_) are defined as,

9 *E*_r_ = *E*_f_ − *E*_i_

where *E*_f_ is enthalpy of final state (product) and *E*_i_ is enthalpy of initial state (reactant).

*E*_a_ can be calculated as follows,

10 *E*_a_ = *E*_T_ − *E*_i_

where *E*_T_ is enthalpy of transition state and *E*_i_ initial states, respectively. Note that, we have used our results of adsorption of ORR intermediates to model the initial and final states of chemical reaction while based on CI-NEB method^57^ as implemented in VASP, the transition states for various ORR elementary steps were located.

The Gibbs free energy of the intermediates were calculated by the following equation:

11 Δ*G*_0_ = Δ*E*_ads_ + ΔZPE − *T*Δ*S*

where Δ*E*_ads_ is calculated from eqn (5)–(8), ΔZPE is the change in zero point energy and Δ*S* the change in entropy at temperature *T*. The zero point energy correction and the entropic contribution are taken from ref. 21. Herein, ΔZPE − *T*Δ*S* is approximated to be 0.4, 0.05 and 0.35 eV for OOH, O and OH adsorbates, respectively. The effect of applied potential (*U*) on the Gibbs free energy can be approximated by

12 Δ*G*(*U*) = Δ*G*(0 V) − *neU*

where *n* number of electrons transferred, *e* elementary positive charge.

The formation energy of the bulk alloy Δ*E*_alloy_ can be estimated as,^41^13
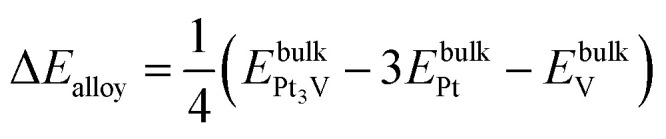


A negative value of the formation energy Δ*E*_alloy_ of the Pt_3_V alloy indicates its thermodynamic stability.

Following the approach in ref. 16 and 55, the extent of the dissolution of Pt from Pt_3_V(111) surface relative to that of pure Pt(111) surface could be estimated with the electrode potential shift (Δ*U*) for reaction Pt → Pt^2+^ + 2e^−^,14
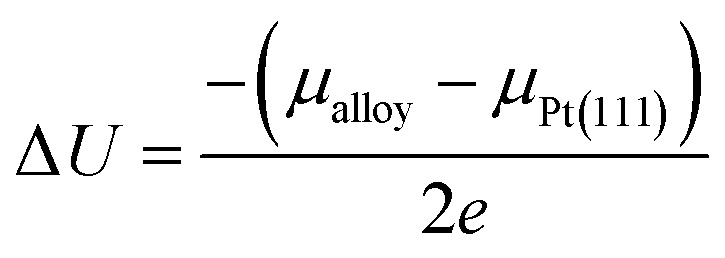
where *μ*_alloy_ and *μ*_Pt(111)_ are the chemical potentials of Pt atoms on the alloy and Pt(111), respectively. Herein, *μ* is calculated as the energy difference between the surface without and with a Pt atom removed from the outermost surface layer. Δ*U* > 0 indicates that the dissolution of Pt would take place at a higher electrode potential, which corresponds to better stability.

## 3 Results and discussion

### 3.1 Surface adsorption sites

Similar to previous works^47,58–61^ four different adsorption sites on pure Pt(111) surface are considered in this study, namely, face centered cubic (fcc), hexagonal close packed (hcp), bridge and top sites. Moreover, due to the presence of V atom in the second layer of Pt_3_V(111) surface, the four adsorption sites on Pt(111) are then further divided into different kinds of adsorption sites, as shown in Fig. 1b.^16,41^ F_1_ denotes a fcc site coordinated above a Pt atom in the third layer, while F_2_ on top of the third layer V. H_1_ is an hcp site above a V atom in the second layer while H_2_ has a Pt atom underneath. T_1_ stands for a top site with one V and two Pt atoms neighbor in the second layer while T_2_ corresponds to the other top site with three Pt atoms neighbor in the second layer. A bridge site denoted as B_1_ is located between T_1_ sites near a V atom in the second layer. Whereas B_2_ is between T_1_ and T_2_ sites. Moreover, B_3_ is a bridge site between T_1_ sites which have a Pt atom neighbor in the second layer.

### 3.2 Adsorption energies of ORR intermediates

The adsorption energies of the ORR intermediates were determined, where only a single molecule of the adsorbate is adsorbed on the surface of the catalyst. Thus, we determined the optimized structures and the binding energy of the basic ORR intermediates (O, OH, and OOH) adsorbed on possible surface sites on the Pt-skin Pt_3_V(111) surface. Likewise to the previous works, this study also considered only the adsorption of O on fcc site, OH on top/bridge sites, and OOH on top site.^16^

**Fig. 2:**
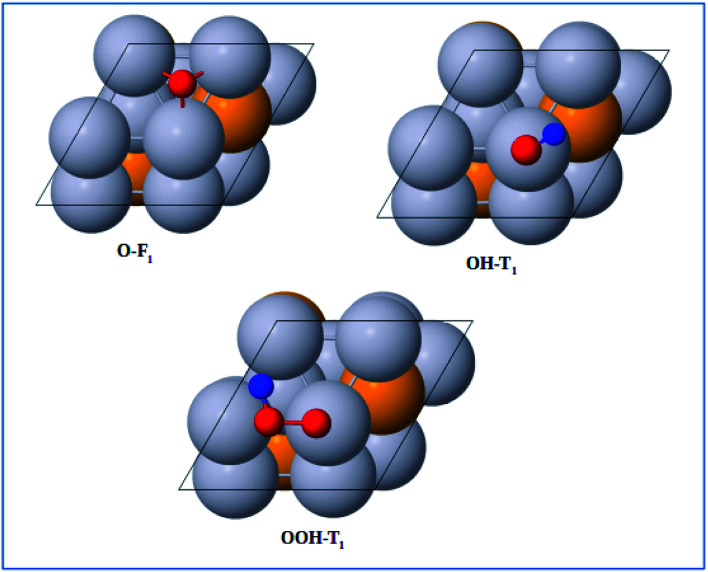
Top view of optimized structures of the lowest-energy configuration for ORR intermediates on a Pt-skin Pt_3_V(111) surface. Pt, V, O, and H atoms are labeled in gray, orange, red and blue, respectively.

The optimized adsorption configurations of the three ORR intermediates are shown in Fig. 2. The adsorption energies (*E*_ads_) of the ORR intermediates are calculated using eqn (5)–(7) at various adsorption sites are presented in Table 1.

**Table 1:** Calculated adsorption energies (Δ*E*_ads_) of ORR intermediates on various adsorption sites of Pt skin Pt_3_V(111) surface. All results are in units of eV

Intermediates	Adsorption energies (Δ*E*_ads_)						
	F_1_	F_2_	T_1_	T_2_	B_1_	B_2_	B_3_
O	2.06	2.40					
OOH			3.93	4.03			
OH			0.75	0.76	0.98	1.06	0.94

As can be seen in Table 1, the most energetically favorable site of O is F_1_ and T_1_ is for both OH and OOH with adsorption energies of 2.06, 0.75 and 3.93 eV, respectively.

**Fig. 3:**
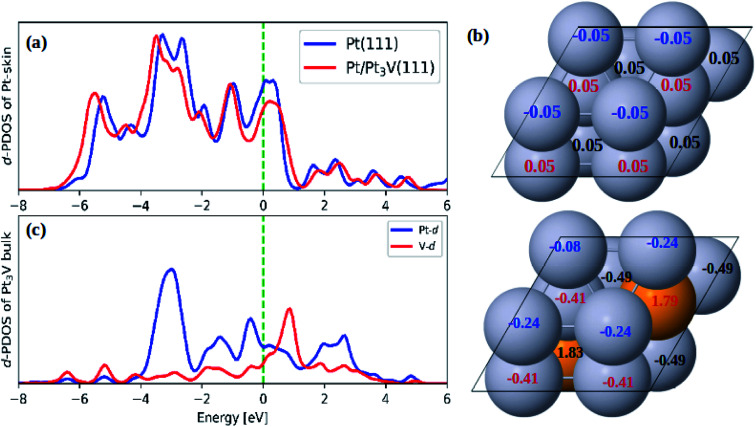
(a) The d-PDOS plots of Pt skin on Pt(111) and Pt-skin Pt_3_V(111); (b) the Bader charge analysis on Pt(111) and Pt-skin Pt_3_V(111); (c) the d-PDOS plots of Pt_3_V bulk. Pt and V atoms are labeled in gray and orange, respectively.

As compared to pure Pt(111) surface, the adsorption energies of ORR intermediates on Pt-skin Pt_3_V(111) are lower (Table 2). This implies the ORR intermediates would interact more weakly on Pt-skin Pt_3_V(111) than on the pure Pt(111) surface. In general, the adsorption property of transition metals can be describe using d band model.^62^ The stronger interaction between the adsorbate and surface is due to an upshift of the antibonding orbitals caused by an upshift of the d band center to the Fermi energy. However, the d band center of Pt-skin on Pt_3_V(111) is downshifted away from the Fermi energy compared to pure Pt(111) Fig. 3a, which is responsible for the weak adsorption of Pt-skin Pt_3_V(111).^62^ It is plausible that the electrostatic interaction also plays an important role in tuning the adsorption ability. Based on a Bader charge analysis each platinum atom withdraws, on average, 0.41e^−^ from V atoms. Furthermore, the Pt-skin of Pt_3_V(111) is more negatively charged compared to pure Pt(111) (Fig. 3b), leading to the stronger electrostatic repulsion between O-containing intermediates and the surface of the catalysts, which is consistent with the weaker adsorption ability. Thus, Pt-skin Pt_3_V(111) possesses the weakest adsorption energies between the two systems.

**Table 2:** Calculated adsorption energies of ORR intermediates on Pt/Pt_3_V(111), Pt(111), and Pt(111) strain surface. Pt(111) strain denotes the strained Pt(111) with the lattice parameter of 3.93 Å. All results are in units of eV

Intermediates	Adsorption energies (Δ*E*_ads_)		
	Pt_3_V(111)	Pt(111)	Pt(111) strain
O	2.06	1.47	1.75
OOH	0.75	0.50	0.78
OH	3.93	3.68	3.97

As demonstrated by previous reports, adsorption ability of the Pt surface can be affected by both the strain effect (caused by the alloying) and ligand effect (due to vanadium atom underneath).^19–21^ In order to distinguish the strain effect from the ligand effect, the strained Pt(111) surface with the lattice parameter of Pt_3_V was constructed. Herein, the adsorption ability of Pt(111) is used as the reference to calculate the change in adsorption energies presented in Table 2. The Δ*E*_ads_ of the strain Pt(111) are 0.28 eV for each of ORR intermediates (OOH, O, and OH). For Pt-skin Pt_3_V(111), the corresponding Δ*E*_ads_ are 0.24, 0.6, and 0.25 eV. Thus, compared with the Δ*E*_ads_, it is found that the weak adsorption ability of Pt-skin Pt_3_V(111) for OOH and O is mainly due to both strain and ligand effect; for OH adsorption, the strain effect is dominant. Therefore, the result revealed that, both the strain and ligand effects could tune adsorption ability of Pt-skin Pt_3_V(111).

### 3.3 Activation and reaction energy of ORR elementary steps

**Fig. 4:**
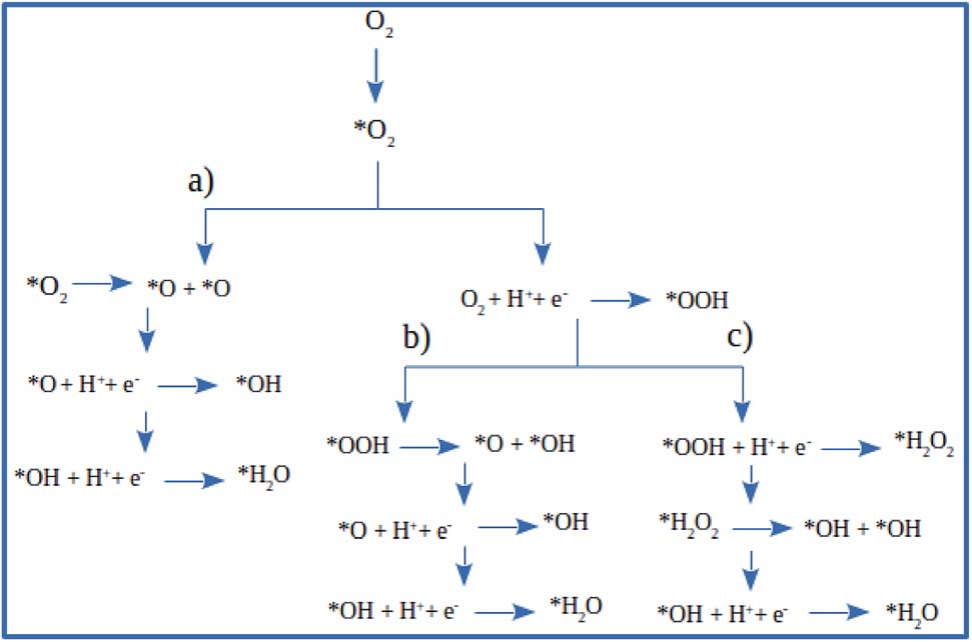
The schematic of ORR reaction mechanisms on Pt-skin Pt_3_V(111): (a) O_2_ dissociation ORR mechanism, (b) OOH dissociation ORR mechanism and (c) H_2_O_2_ dissociation ORR mechanism.

There are three possible ORR mechanisms. These are O_2_ dissociation mechanism, OOH dissociation mechanism, and H_2_O_2_ dissociation mechanism. The ORR mechanisms elementary steps include O_2_ dissociation, OOH formation, OOH dissociation, OH formation, H_2_O_2_ formation, H_2_O_2_ dissociation, and H_2_O formation^16,41,63,64^ as shown in Fig. 4. The reaction energies (*E*_r_) and activation energies (*E*_a_) were calculated for all elementary steps of the three ORR mechanisms using eqn (9) and (10), respectively. Results are presented in Table 3.

**Table 3:** The activation energies (*E*_a_) and reaction energies (*E*_r_) for elemental steps in ORR. The values in parenthesis are taken from ref. 41. All results are in unit of eV

Reaction steps	Pt-skin Pt_3_V(111)		Pt(111)	
	*E*_r_	*E*_a_	*E*_r_	*E*_a_
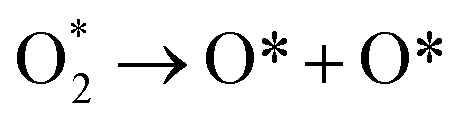	−0.41	1.00	−0.75(−0.88)	0.77(1.02)
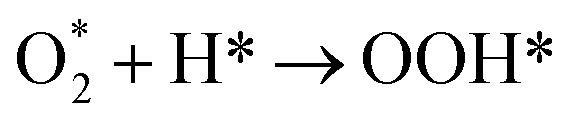	−0.11	0.36	−0.08(−0.21)	0.33(0.44)
OOH* → O* + OH*	−1.20	0.33	−1.43(−1.49)	0.23(0.22)
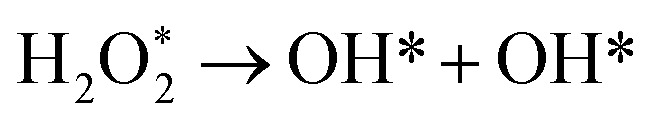	−1.44	0.20	−1.65(−1.75)	0.14(0.26)
O* + H* → OH*	−0.60	0.64	−0.27(−0.19)	0.72(0.97)
OH* + H* → H_2_O*	−1.07	0.29	−0.73(−0.75)	0.12(0.18)
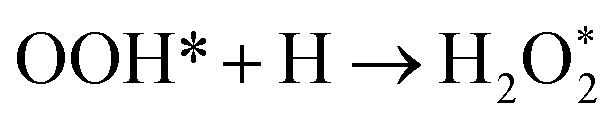	−0.49	0.42	−0.19(−0.28)	0.37(0.19)

#### 3.3.1 Dissociation reactions

**Fig. 5:**
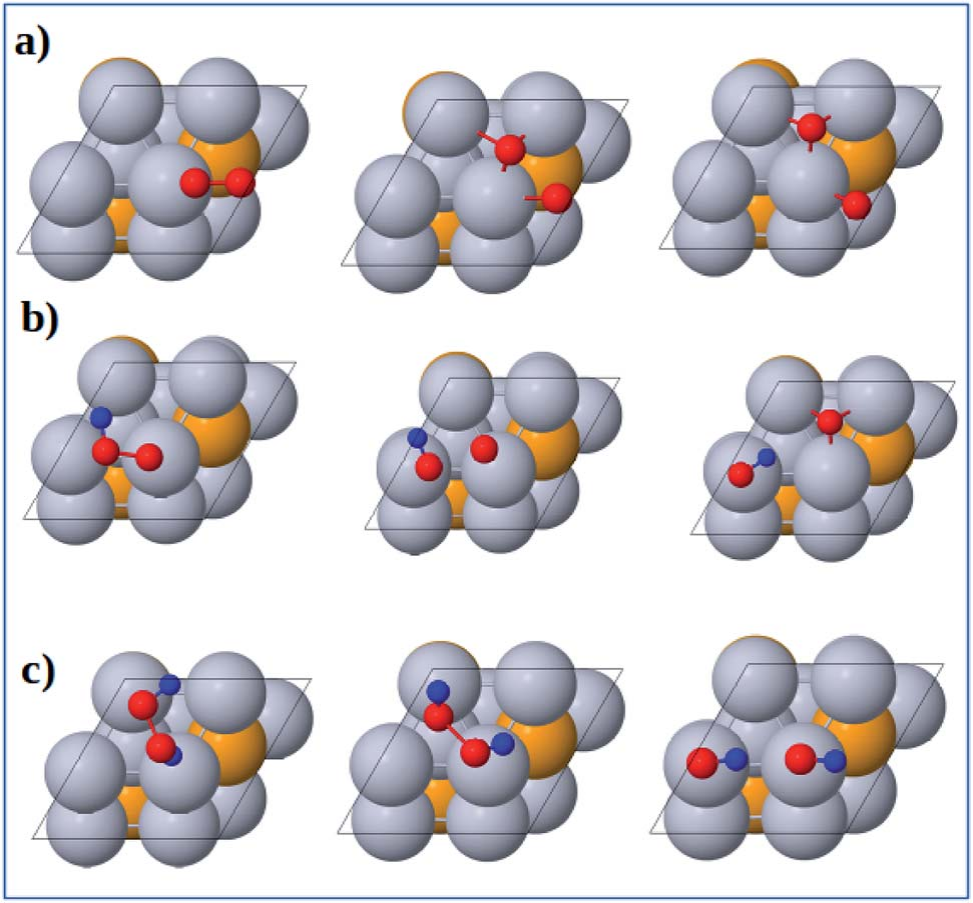
Atomic structures of the initial state (left figure), transition state (middle figure), final state (right figure) for (a) O_2_ dissociation, (b) OOH dissociation and (c) H_2_O_2_ dissociation reactions on the Pt-skin Pt_3_V(111) surface. Pt, V, O and H atoms are labeled in gray, orange, red and blue, respectively.

**Fig. 6:**
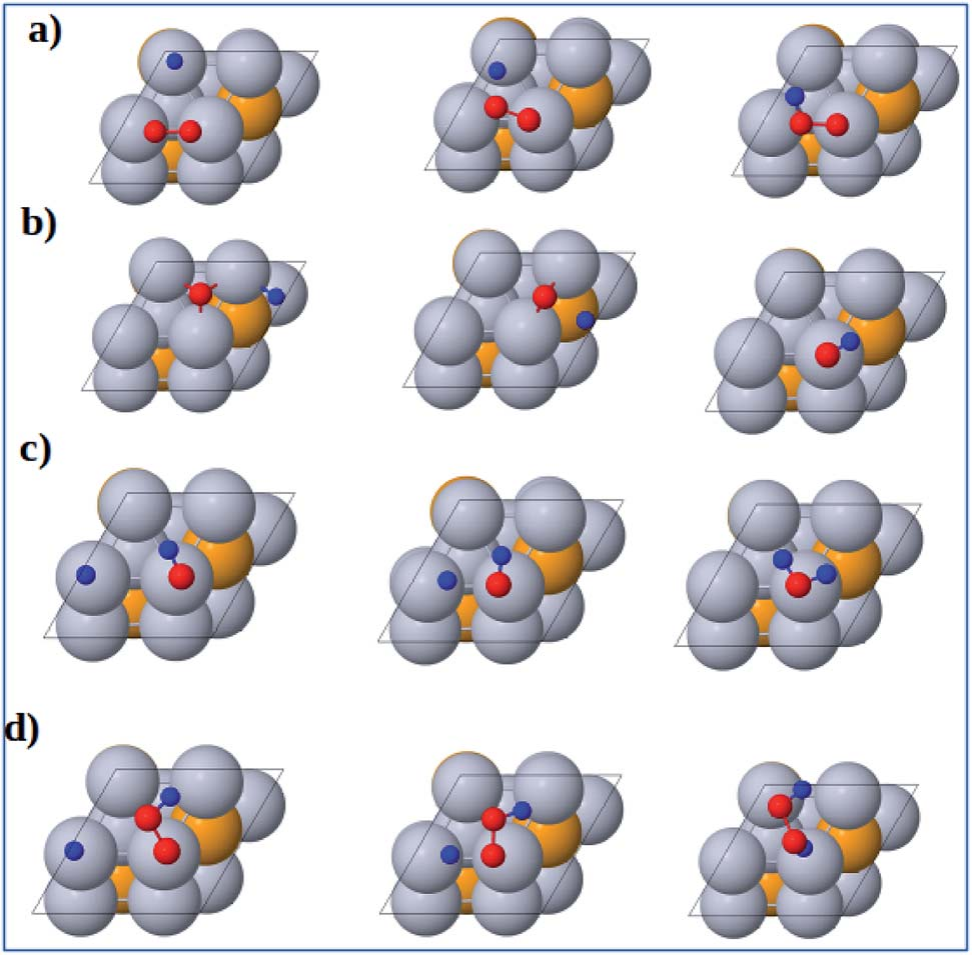
Atomic structures of the initial state (left figure), transition state (middle figure), and final state (right figure) for (a) OOH formation, (b) OH formation, (c) H_2_O formation, and (d) H_2_O_2_ formation reactions on the Pt_3_V(111) surface. Pt, V, O and H atoms are labeled in gray, orange, red and blue, respectively.

The optimized geometries of the initial, transition and final states for ORR elementary reactions are given in Fig. 5 and 6. The initial, transition and final states of O_2_ dissociation reaction are shown in Fig. 5a. The initial and final states are the adsorption of O_2_ at B_1_ and the coadsorption of two O atoms at the two adjacent F_1_ sites, respectively. In the transition state, one O atom stays at the bridge site while the other O atom moves toward an F_1_ site.

The initial, transition and final states of OOH dissociation reaction are shown in Fig. 5b. The initial and final states of this reaction are the adsorption of OOH at T_1_ and the coadsorption of OH at the T_1_ sites and O at an F_1_ site, respectively. In the transition state, the O–O bond is broken and then the separated O and OH move towards F_1_ and T_1_ sites, respectively.

The initial, transition and final sates of H_2_O_2_ dissociation reaction are shown in Fig. 5c. The initial state is the adsorption of OOH at the B_2_ site while the final state is the coadsorption of the separated OH molecules at the two T_1_ sites. In the transition state, O–O bond is elongated and the two OH groups rotate. The rotation of two OH groups causes O–O bond distortion in H_2_O_2_ which further facilitates the O–O bond scission.

#### 3.3.2 Formation reactions

The initial, transition and final states of OOH formation reaction are shown in Fig. 6a. The initial state is the coadsorption of O_2_ at B_3_ and H at T_2_ and the final state is the adsorption of OOH at T_1_ site. In the transition state, the O_2_ and H get closer to each other to form OOH.

The initial, transition and final states of OH formation reaction are shown in Fig. 6b. In this reaction the initial state is the coadsorption of H and O at the two adjacent F_1_ site, and the final state is the adsorption of OH at a bridge T_1_ site. In its transition state, O atom moves to bridge B_1_ site and H also moves toward O.

The initial, transition and final states of H_2_O formation reaction are shown in Fig. 6c. The initial state of this reaction is the coadsorption of OH at T_1_ site and H at the nearby T_1_ site, and the final state is the adsorption of H_2_O at T_1_ site. In the transition state, the OH and O get closer to each other to form H_2_O.

The initial, transition and final states of H_2_O_2_ formation reaction are shown in Fig. 6d. The initial state of this reaction is the coadsorption of OOH at T_1_ site and H at the nearby T_1_ site, and the final state is the adsorption of H_2_O_2_ at a B_2_ site. In the transition state, H atom move towards OOH to form H_2_O_2_.

### 3.4 ORR mechanisms on the Pt-skin Pt_3_V(111) surface

The first step for ORR is the adsorption of O_2_, which proceeds *via* direct O_2_ dissociation or OOH formation by hydrogenation. The *E*_a_ would help as the selection criteria between O_2_ dissociation and OOH formation.^41^ As shown in Table 3, O_2_ dissociation exhibited much higher activation energy (1 eV) than that of OOH formation (0.36 eV). Therefore, OOH formation is the preferred initial step for ORR. After OOH formed, it splits into O and OH with the activation energy of 0.33 eV; if not, H_2_O_2_ could be formed and later dissociate into 2OH with an activation energy of 0.20 eV. Therefore, the O–O bond cession reaction is taking place through OOH dissociation. Similarly, there is another reaction step that leads to the formation of OH, which is, the hydrogenation of O result in OH formation with an activation energy of 0.64 eV. For H_2_O formation, the *E*_a_ of OH reacts with H is 0.29 eV. The last step of ORR is H_2_O desorption and recovery of the surface of catalysts, which needs to overcome 0.29 eV barrier. According to the above analysis, the ORR mechanism on Pt-skin Pt_3_V(111) is OOH dissociation mechanism, as summarized in the following:O_2_ adsorption (no barrier)OOH formation: O_2_ + H → OOH (*E*_a_ = 0.36 eV)OOH dissociation: OOH → O + OH (*E*_a_ = 0.33 eV)O hydrogenation: O + H → OH (*E*_a_ = 0.64 eV)H_2_O formation: OH + H → H_2_O (*E*_a_ = 0.29 eV)

The result suggested that the dissociation of OOH would be the preferred ORR mechanism on Pt-skin Pt_3_V(111) surface. Moreover, the study revealed that, O hydrogenation have the highest activation energy (0.64 eV) therefore, OH formation reaction is the rate-determining step for ORR. According to results, O hydrogenation reaction with the activation energy of (0.72 eV) was found to be the rate-determining step for the favorable OOH dissociation ORR on pure Pt(111) surface. As a result, comparing the activation energy of the rate determining steps of ORR on Pt and Pt-skin Pt_3_V(111) surface, it is found that the activation energy on Pt-skin Pt_3_V(111) is much lower than that of Pt(111) surface as presented in Table 3. Thus, ORR on Pt-skin Pt_3_V(111) surface can taking place easily.

### 3.5 Gibbs free energy diagram

Since Pt-skin Pt_3_V(111) is promising for fuel cell applications, it is necessary to explore the thermodynamics of the cathode reaction for Pt-skin Pt_3_V(111). In order to investigate a complete cathode reaction pathway, we will focused on the reaction pathways represented by associative mechanism eqn (15).15a
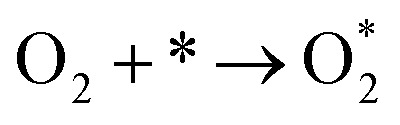
15b
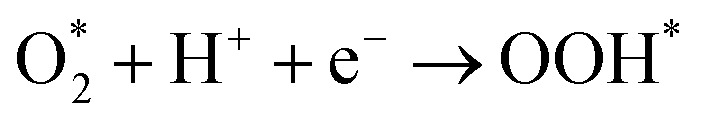

15c OOH* + H^+^ + e^−^ → O* + H_2_O

15d O* + H^+^ + e^−^ → OH*

15e OH* + H^+^ + e^−^ → H_2_O + *

where * denotes an active site on the catalyst. According to eqn (15a)–(15e), the reaction Gibbs free energy can be written as

16a * + O_2_ + H^+^ + e^−^ → *OOH, Δ*G*_1_ = Δ*G*_ads_(OOH*) − 4.92 eV

16b *OOH + H^+^ + e^−^ → *O + H_2_O, Δ*G*_2_ = Δ*G*_ads_(O*) − Δ*G*_ads_(OOH*)

16c *O + H^+^ + e^−^ → *OH, Δ*G*_3_ = Δ*G*_ads_(OH*) − Δ*G*_ads_(O*)

16d *OH + H^+^ + e^−^ → H_2_O + *, Δ*G*_4_ = −Δ*G*_ads_(OH*)

**Fig. 7:**
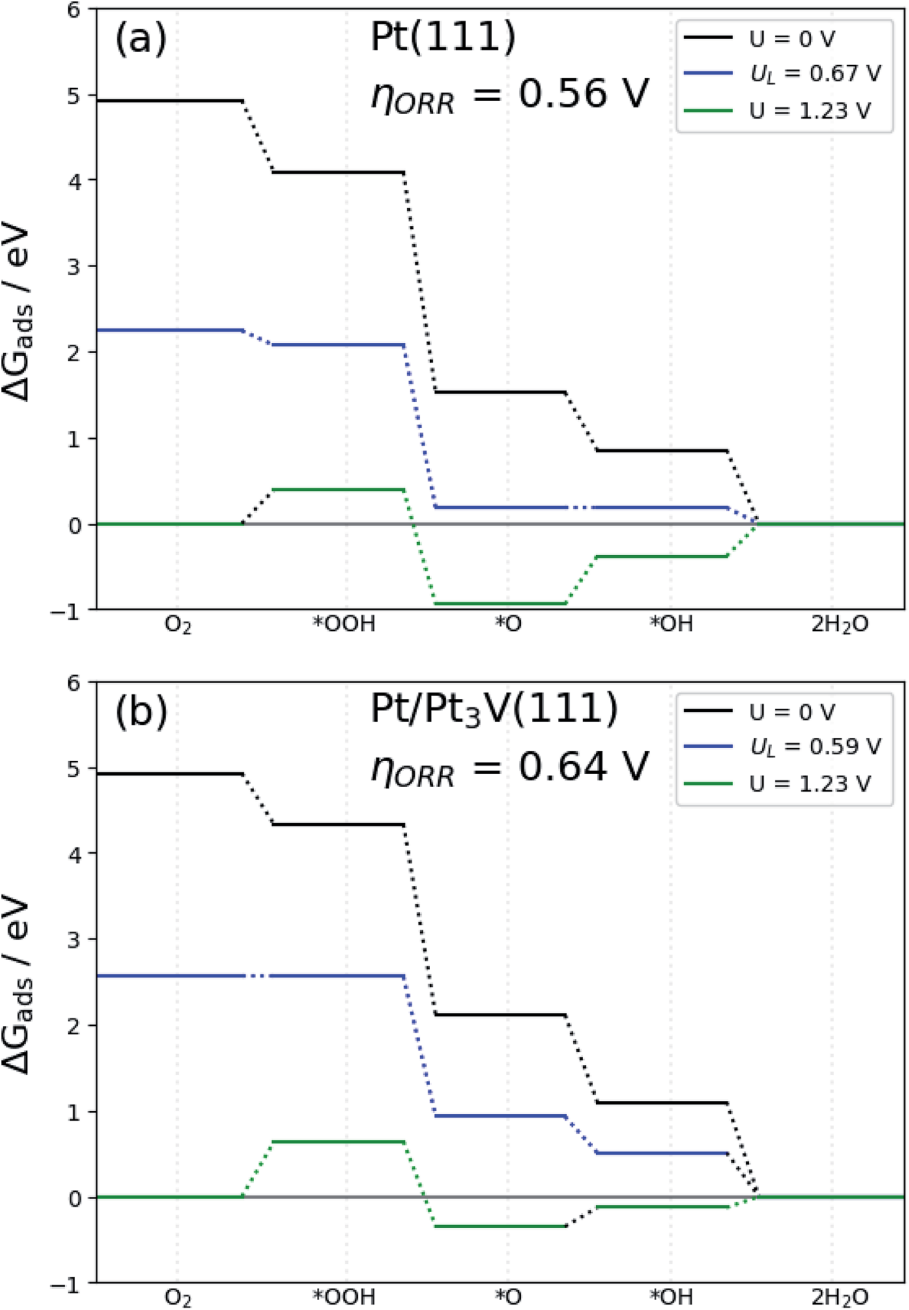
Free-energy diagram for oxygen reduction on: (a) Pt(111) and (b) Pt-skin Pt_3_V(111). Results are shown at zero cell potential (*U* = 0), at the equilibrium potential (*U* = 1.23 V), and at the limiting potential (*U*_L_) where all reaction steps are exothermic.

The free energy diagram for OOH dissociation mechanism at different electrode potential are presented in Fig. 7b. The free energy diagram shows that with the electrode potential (*U* = 0 V), all the elementary steps of the ORR on the Pt-skin Pt_3_V(111) surfaces are exothermic (negative free energy changes). When the electrode potential increase the hydrogenation reaction become less negative and thus, at equilibrium potential *U* = 1.23 V, the O hydrogenation reactions are endothermic on the Pt-skin Pt_3_V(111) surfaces. The limiting potential of the electrocatalysts is define interms of this equilibrium potential. The limiting potential is the highest potential at which the whole reaction path is exergonic. In light of Fig. 7, the highest electrode potential is 0.59 V with the ORR progression *via* the OOH dissociation mechanism. The calculated ORR overpotential for Pt-skin Pt_3_V(111) (Fig. 7b) is 0.64 V with limiting step of OOH formation, which is slightly higher than for the Pt(111) surface (Fig. 7a).

### 3.6 Stability of Pt_3_V alloy

The stability of Pt_3_V alloy may be assessed with the corresponding alloy formation energy (Δ*E*_alloy_) and the electrode potential shift, Δ*U*. The formation energy was calculated using eqn (13) and the corresponding alloy formation energy is found to be −0.36 eV per atom, which is comparable with formation energy of Pt_3_V (which is between −0.4 and −0.3 eV) and Pd_3_V (−0.3 eV).^13,65^ Thus, the negative value of formation energy indicates the better stability of Pt_3_V alloy.

**Fig. 8:**
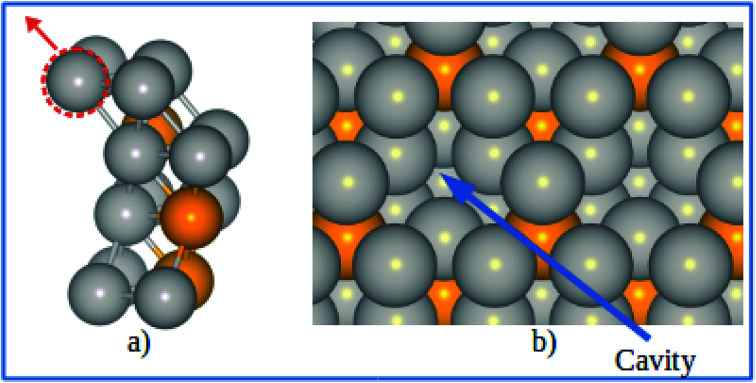
(a) Schematics of our atomic model for evaluating the change of chemical potentials. (b) Top view of the cavity positions on the Pt-skin surface. Pt and V atoms are labeled in gray and orange respectively.

The electrode potential shift Δ*U* were obtained from eqn (14). As shown in Fig. 8, for the alloy systems, the top most slab layer contains four Pt atoms while the sublayer has three Pt atoms and one V atom each in the unit cell. Removing one Pt atom from the surface produces a cavity structure (Fig. 8). Due to the V atoms in the subsurface, the total energy of slab with cavity varies with the position of the removed Pt atom. The chemical potentials were obtained from the configuration with the lowest total slab energy. Our results clearly show the stability enhancement of Pt-skins: our DFT calculated value of the electrode potential shift is 0.055 V. The positive values of the shifts indicate that the dissolution of Pt atoms from Pt-skin surfaces are occurring at higher potentials.

**Fig. 9:**
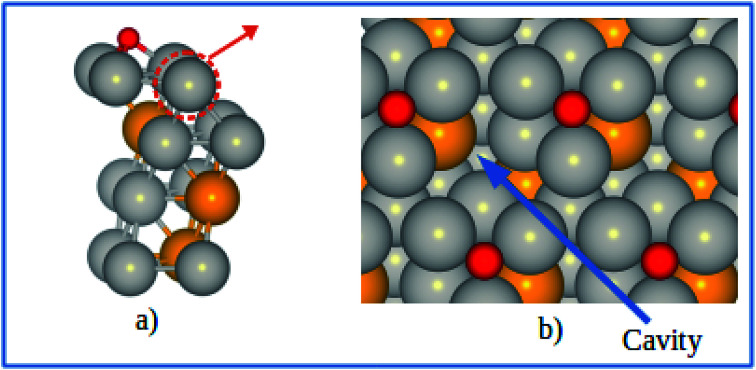
(a) Schematics of our atomic model for describing the Pt-skin structure with oxygen adsorption at the fcc sites. (b) Top view of the oxygen adsorption sites and cavities on the skin surface. Pt, V, and O atoms are labeled in gray, orange, and red respectively.

However, a more realistic surface is that where oxygenated species are adsorbed as a consequence of the catalyzed reaction. In the ORR, the adsorption of various intermediates may affect the stability of Pt and Pt-based alloy catalysts. The approach described above is capable of estimating the electrochemical stability and the dissolution trend in the presence of adsorbates. Similar slab model was used to investigate the potential shift of pure Pt and Pt-skins under 0.25 ML of adsorbed atomic oxygen. The most stable adsorption sites (fcc) for the Pt-skin surfaces are shown in Fig. 9.

Generally speaking, Pt dissolution becomes easier from an oxygenated surface compared to a clean surface. For the pure Pt(111) surface, it is predicted that the electrode potential shift (Δ*U*) is found be −0.375 V between an oxygenated and a clean surface, which is in good agreement with previous theoretical result of Δ*U* = −0.360 V for the Pt(111) surface.^16,41^ Similarly, an electrode potential shift of Δ*U* = −0.365 V is obtained for an oxygenated Pt-skin Pt_3_V(111) surface relative to the clean Pt-skin Pt_3_V(111) surface. Comparing the oxygenated Pt-skin Pt_3_V(111) and Pt(111) surfaces, Δ*U* found to be 0.065 V, which suggests that Pt-skin Pt_3_V(111) electrocatalyst would have better stability against surface Pt dissolution than the pure Pt electrocatalyst even under the oxidizing electrochemical conditions.

## 3.7 Conclusions

Density functional theory analysis was employed to investigate the catalytic performance of Pt-skin Pt_3_V(111) electrocatalyst for ORR in PEMFCs. The results showed that the ORR intermediates (O, OH and OOH) are weakly adsorbed on Pt-skin Pt_3_V(111) compared to pure Pt(111). Moreover, the ORR mechanism of Pt-skin Pt_3_V(111) procced *via* the OOH dissociation mechanism and the rate determining step is the formation of OH with an activation energy of 0.64 eV. The negative formation energy of the Pt_3_V (111) alloy (*i.e.*, −0.36 eV per atom) and the positive dissolution potential shift of the surface Pt atoms revealed the better stability of Pt-skin Pt_3_V(111) surface with and without O adsorption compared with pure Pt(111) surface. Therefore, Pt-skin Pt_3_V(111) alloy can have not only better catalytic activity but also good stability for ORR in PEMFCs as compared to the pure Pt(111) electrocatalyst.

## Conflicts of interest

There are no conflicts to declare.

## Supplementary Material
